# Competitiveness and individual characteristics: a double-blind placebo-controlled study using oxytocin

**DOI:** 10.1038/s41598-020-68445-w

**Published:** 2020-07-13

**Authors:** Hirofumi Kurokawa, Yusuke Kinari, Hiroko Okudaira, Kiyotaka Tsubouchi, Yoshimichi Sai, Mitsuru Kikuchi, Haruhiro Higashida, Fumio Ohtake

**Affiliations:** 1 School of Economics and Management, University of Hyogo, 8-2-1 Gakuen-nishi-machi, Nishi-ku, Kobe, Hyogo 651-2197 Japan; 2 Hirao School of Management, Konan University, Nishinomiya, Hyogo Japan; 3 Doshisha Business School, Doshisha University, Kyoto, Kyoto Japan; 4 Department of Biophysical Genetics, Research Center for Child Mental Development, Kanazawa University, Kanazawa, Japan; 5 Department of Economics, Osaka University, Toyonaka, Osaka Japan

**Keywords:** Social behaviour, Social neuroscience, Hormones

## Abstract

Oxytocin-enhanced prosocial behaviour depends on individual characteristics. This study investigated the relationship between oxytocin and competitiveness, which is another important social trait and predicts economic and social outcomes. In this double-blind, randomized, and placebo-controlled study of 192 male participants, we examined whether oxytocin moderates competitiveness and whether the effect of oxytocin on competitiveness is amplified in individuals with autistic traits. While our results show no relationship between oxytocin and competitiveness, we observed suggestive patterns: albeit not significantly, oxytocin reduced and enhanced competitiveness among participants without autistic traits and among their counterparts with autistic traits, respectively.

## Introduction

While oxytocin reportedly enhances prosocial behaviours such as trust^1^, generosity^2^, and parochial altruism^3^, recent studies have shown this effect to depend on context and individual characteristics^4^ like autistic traits^5^ and baseline oxytocin levels^6^. Hirosawa et al.^5^ found that oxytocin induces prosocial behaviours in individuals with traits associated with autism spectrum disorder (ASD)^7^—i.e., low empathy quotient (EQ)^8^, high systemizing quotient (SQ)^9^, and high autism spectrum quotient (AQ)^10^ scores^11–13^. It is important to investigate for whom and in what aspects oxytocin can be effective, because oxytocin has clinical implications for people with ASD, who commonly show defects in social behaviour^14^.

The current study expands the literature on the behavioural effects of oxytocin in two ways. First, we tested whether oxytocin moderates the scantly investigated but important social trait of competitiveness; while the oxytocin literature shows a relative dearth concerning the potential effect of oxytocin on competitiveness, recent social science research finds that competitiveness can predict important economic and social outcomes^15–17^. Second, we tested whether the effect of oxytocin is amplified in individuals with autistic traits.

Experimentally, competitiveness can be measured according to a paradigm developed by Niederle and Vesterlund^18^: individuals choose a payment scheme between a piece-rate and a tournament environment. While the piece-rate environment is non-competitive, the tournament environment is competitive because one’s payment depends on relative group performance. The tournament represents labour market outcomes, such as promotions and hiring. A growing body of literature focuses on the effects of external factors such as environment^19–21^, culture^22,23^, and internal factors such as hormones^24–27^ and preferences^28,29^ on competitiveness. Bartling et al.^28^ found that individuals with higher personality trait scores for “agreeableness” were less likely to enter tournaments than to enter piece-rate schemes. These findings suggest a negative association between competitiveness and agreeableness, which has been found to relate to cooperative preferences^30^ and prosocial behaviours, including trust and altruism^31^. Additionally, a seminal study by Kosfeld et al.^1^ showed that oxytocin increases prosocial behaviours; namely, oxytocin increased an investor’s transfer to a trustee during a trust game^1^ as well as parochial altruism^3^. The observed effects of oxytocin on prosocial behaviour indicate a possible inhibitory influence of oxytocin on an individual’s preference for competition.

Deficits in social behaviour is a diagnostic feature of ASD. Although, the existence of low plasma oxytocin levels in autism are still controversial^32^ and the effects of oxytocin on ASD is still uncertain, oxytocin may be a suitable candidate substance for improving the sociality of ASD^33^. Intriguingly, previous studies have demonstrated that the effect of oxytocin on prosocial behaviours was stronger among individuals with autistic-like traits^5^ and among individuals with ASD exhibiting low baseline endogenous plasma oxytocin^6^. These observations have informed the hypothesis that oxytocin exerts a stronger effect on competitiveness in individuals with more autistic traits than in their counterparts without them. To assess this possibility, we measured competitiveness following the administration of oxytocin to individuals with and without autistic traits.

## Results

### Effects of oxytocin on competitiveness

One hundred ninety-two male participants were randomly assigned to two groups receiving either oxytocin or a placebo. After administration, we conducted a competition experiment (CE) modelled after the framework used by Niederle and Vesterlund^18^ (see “Methods”). In this CE, participants first performed a simple task under a piece-rate scheme (Task 1): they received ¥50 for every point. Second, participants performed the task under a tournament scheme (Task 2): the individual who earned the most points in each group received ¥200 for every point, while the others received ¥0. In the third task, the participants could choose whether to participate in the piece-rate and tournament schemes (Task 3). The selection of the tournament scheme indicated the participant’s preference for competition.

**Figure 1:**
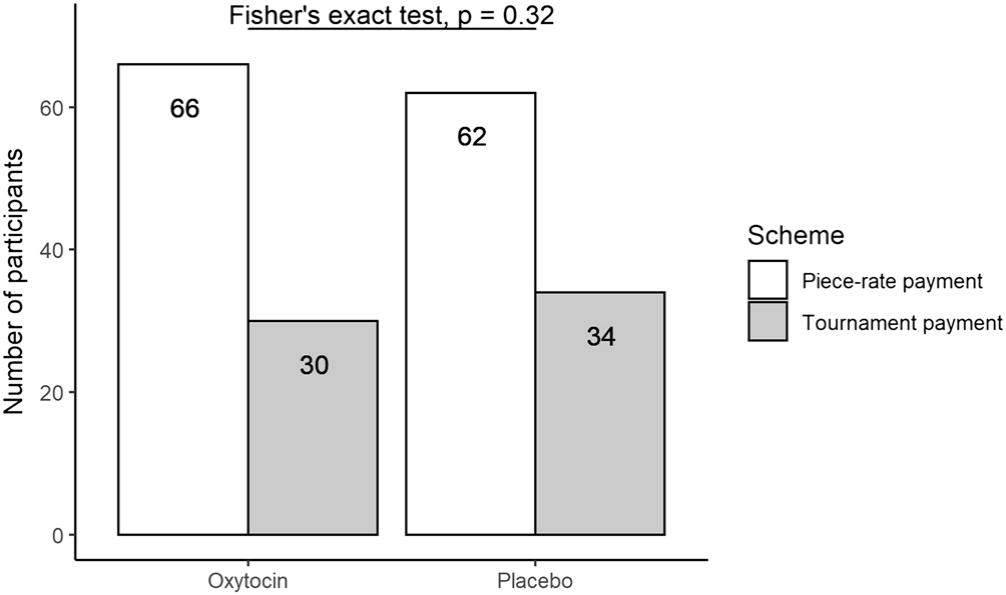
Number of participants who selected the piece-rate or tournament payment scheme in Task 3 in the oxytocin and in the placebo group. Thirty of the 96 participants in the oxytocin and 34 of the 96 participants in the placebo group chose the tournament payment scheme (Fisher’s exact test: *p* = 0.32, one-sided, Odd’s ratio = 0.83).

Figure 1 shows the number of participants that selected the tournament in Task 3: 30 of 96 participants (31.2%) in the oxytocin group, and 34 of 96 participants (35.4%) in the placebo group. There was no statistically significant difference between the numbers and percentages of participants in the two groups who selected the tournament (Fisher’s exact test, *p* = 0.32, one-sided, Odd’s ratio = 0.83; *t* = − 0.61, *p* = 0.27, one-sided, 95% confidence interval (CI) = − 0.93 to − 0.18, Cohen’s *d* = 0.09).

### Heterogenous effects of oxytocin on competitiveness

Although we found no effect of oxytocin on competitiveness among participants at baseline, we considered the possibility that oxytocin might exert heterogenous effects that depend on contextual and individual characteristics, including autistic-like traits^4–6^. For example, the effect of oxytocin has been found to be stronger among individuals with autistic-like traits^5^. We thus investigated whether oxytocin moderated competitive behaviours in participants who exhibit autistic-like traits; these individuals were identified according to their scores on the AQ^10^, EQ^8^, and SQ^9^ questionnaires administered on the day before the start of the experiment.

Table 1 summarizes the results of the statistical analyses. There were no significant differences in AQ (*t* = − 0.04, *p* = 0.97, two-sided), EQ (*t* = 0.02, *p* = 0.98, two-sided), and SQ (*t* = − 0.20, *p* = 0.84, two-sided) scores between the oxytocin and placebo groups. Supplement Table 1 also shows no significant differences in the AQ subscale scores between the oxytocin and placebo groups (Social skills: *t* = − 0.15, *p* = 0.88, two-sided; Attention switching: *t* = − 0.22, *p* = 0.83, two-sided; Attention detail: *t* = − 0.45, *p* = 0.65, two-sided; Communication: *t* = − 0.03, *p* = 0.97, two-sided; Imagination: *t* = − 0.07, *p* = 0.56, two-sided;). To generate a variable for autistic-like traits, we generated a dummy variable, AQ(H), which equalled 1 if the AQ score was higher than the median.

**Table 1:** Summary statistics.

	All			Oxytocin			Placebo			*p*-values
	N	Mean	SD	N	Mean	SD	N	Mean	SD	
AQ	192	20.22	6.67	96	20.20	6.38	96	20.24	6.98	0.97
EQ	190	16.64	7.38	94	16.65	7.10	96	16.63	7.68	0.98
SQ	189	19.20	9.80	93	19.05	9.60	96	19.34	10.04	0.84
AQ(H)	192	0.46	0.50	96	0.42	0.50	96	0.51	0.50	0.19
Component 2 of EQSQ	189	0.02	0.91	93	0.03	0.91	96	0.00	0.91	0.84
EQ(L)–SQ(H)	189	0.50	0.50	93	0.51	0.50	96	0.49	0.50	0.83
Effort level under piece-rate scheme (Task 1)	192	18.60	4.10	96	18.73	4.48	96	18.47	3.70	0.66
Effort level under tournament scheme (Task 2)	192	19.20	3.91	96	19.35	4.03	96	19.05	3.81	0.59

Neither autistic-like traits nor effort levels show a difference between the oxytocin and the placebo group, indicating that the randomization assignment was successful.

*AQ* autism spectrum quotient, *EQ* empathy quotient, *SQ* systemizing quotient, *AQ(H)* AQ score higher than the median, *EQ(L)–SQ(H)* Low-EQ and High-SQ (the score of the second component is lower than the median), *SD* standard deviation.

As individuals with ASD have relatively high AQ scores^10^, performance on the AQ is used to inform the diagnosis of ASD; furthermore, ASD has been associated with low EQ and high SQ scores^34^. Hence, to assess these traits, we conducted a principal component analysis. The first component indicated a positive EQ and SQ, and the second component indicated a positive EQ and a negative SQ. The first component explained 59.9% of the total variance, while the second explained 40.1%; the first and second components largely explained the total variance. We used the second component, instead of the first, to generate a variable for autistic-like traits on account of the following: First, a higher second component was indicative of a higher EQ and lower SQ (Supplement Fig. 2), and second, the second component negatively correlated with the AQ (Person’s *r* = − 0.29, *p* < 0.01). Therefore, individuals with a higher AQ had a lower second component or a lower EQ and a higher SQ. We generated another dummy variable, EQ(L)–SQ(H), which equalled 1 if the score of the second component was lower than the median. We found no significant differences in AQ(H) (*t* = − 1.30, *p* = 0.19, two-sided) or EQ(L)–SQ(H) (*t* = 0.22, *p* = 0.83, two-sided) between the oxytocin and placebo groups. We also found no significant differences in AQ subscale scores (social skills, attention switching, attention to detail, communication, and imagination) between the oxytocin and placebo groups (Supplement Table 1).

**Figure 2:**
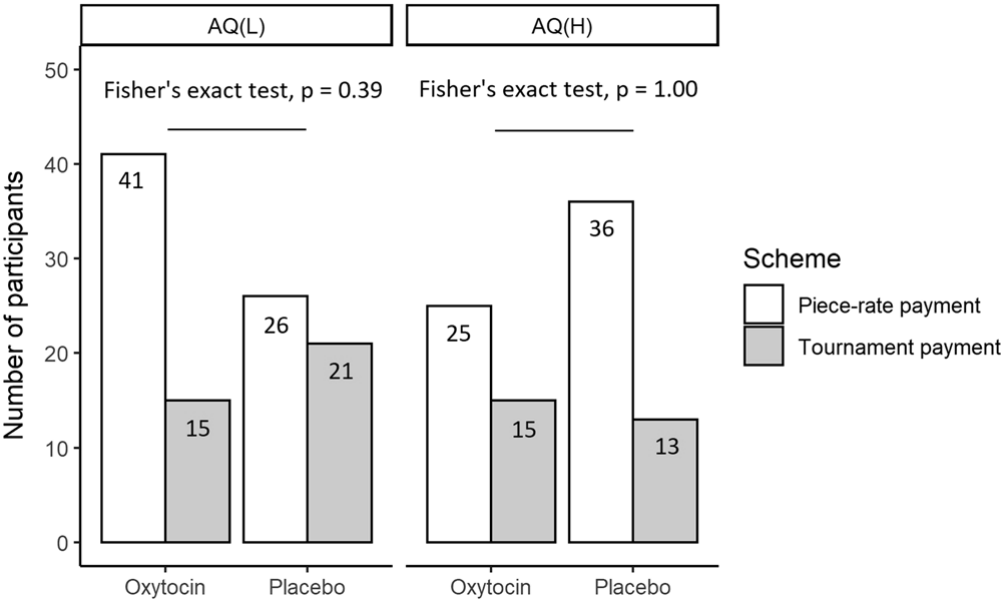
Number of participants who selected the piece-rate or tournament payment scheme in Task 3 according to treatment and autism spectrum quotient (AQ) scores. We assigned participants whose AQ scores were higher and lower than the median into the AQ(H) and the AQ(L) group, respectively. Fifteen of the 56 AQ(L) participants in the oxytocin group and 21 of the 47 AQ(L) participants in the placebo group chose the tournament payment scheme (Fisher’s exact test with *p* values adjusted according to the Benjamini and Hochberg method, *p* = 0.39, Odd’s ratio = 0.45). Fifteen of the 40 AQ(H) participants in the oxytocin group and 13 of the 49 AQ(H) participants in the placebo group chose the tournament payment scheme (Fisher’s exact test with *p*-values adjusted according to the Benjamini and Hochberg method, *p* = 1.00, Odd’s ratio = 1.67).

**Figure 3:**
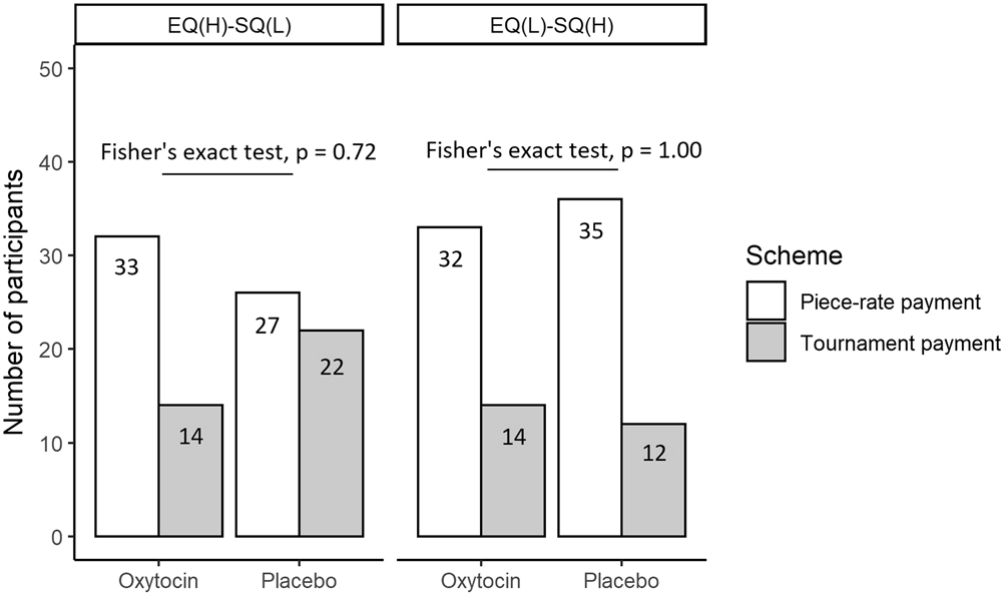
Number of participants who selected the piece-rate or tournament payment scheme in Task 3 according to treatment and empathy quotient (EQ)/systemizing quotient (SQ) scores. We assigned the participants whose scores on the second principal component were lower (i.e., autistic-like traits) and higher than the median into the EQ(L)–SQ(H) and the EQ(H)–SQ(L) group, respectively. Fourteen of the 47 EQ(H)–SQ(L) participants in the oxytocin group and 22 of the 49 EQ(H)–SQ(L) participants in the placebo group chose the tournament payment scheme (Fisher’s exact test with *p* values adjusted according to the Benjamini and Hochberg method; *p* = 0.72, Odd’s ratio = 0.52). Fourteen of the 46 EQ(L)–SQ(H) participants in the oxytocin group and 12 of the 47 EQ(L)–SQ(H) participants in the placebo group chose the tournament payment scheme (Fisher’s exact test with *p* values adjusted according to the Benjamini and Hochberg method; *p* = 1.00, Odd’s ratio = 1.28).

Figures 2 and 3 show the number of participants that entered the tournament in Task 3, according to autistic-like traits. We divided the participants into the AQ(H) and AQ(L) groups according to whether their scores were higher or lower than the median, respectively (Fig. 2). Fifteen of 56 AQ(L) participants (26.8%) in the oxytocin group and 21 of 47 AQ(L) participants (44.7%) in the placebo group entered the tournament. Although it seems that AQ(L) participants in the oxytocin group were less likely to enter the tournament than AQ(L) participants in the placebo group, there was no statistically significant difference between the number and percentage of AQ(L) participants in either group who selected the tournament (Fisher’s exact test with p values adjusted following the Benjamini and Hochberg method, *p* = 0.39, Odd’s ratio = 0.45; *t*-test with *p* values adjusted using Tukey’s method, *p* = 0.22, 95% CI = − 0.06 to 0.42, Cohen’s *d* = 0.38). Fifteen of 40 AQ(H) participants (37.5%) in the oxytocin group and 13 of 49 AQ(H) participants (26.5%) in the placebo group entered the tournament; these proportions did not differ significantly (Fisher’s exact test with *p* values adjusted following the Benjamini and Hochberg method, *p* = 1.00, Odd’s ratio = 1.67 ; *t*-test with *p* values adjusted using Tukey’s method, *p* = 0.69, 95% CI = − 0.37 to 0.15, Cohen’s *d* = -0.24). Supplement Figs. 3–7 show the number of participants that entered the tournament in Task 3 based on each subscale of the AQ. As in the AQ analysis, we divided the participants according to whether their scores were higher or lower than the median. We found subscale patterns, for social skills, attention switching, attention to detail, and imagination, that were similar to the total score pattern: although the differences were not significant, the proportion of participants with lower AQ subscale scores who entered the tournament was lower in the oxytocin group than in the placebo group, and the proportion of participants with higher AQ subscale scores who entered the tournament was higher in the oxytocin group than in the placebo group. The communication subscale showed a different pattern: a smaller (albeit not significantly) proportion of participants entered the tournament in the oxytocin group than in the placebo group, regardless of communication score.

In Fig. 3, we divided the participants into two groups, the EQ(L)–SQ(H) (participants’ scores on the second component were lower than the median—i.e., participants with autistic-like traits) and the EQ(H)–SQ(L) (participants’ scores on the second component were higher than the median) group. We found that 14 of the 47 EQ(H)–SQ(L) participants (29.8%) in the oxytocin group and 22 of the 49 EQ(H)–SQ(L) participants (44.9%) in the placebo group entered the tournament. Although it seems that EQ(H)–SQ(L) participants in the oxytocin group were less likely to enter the tournament than EQ(H)–SQ(L) participants in the placebo group, there was no statistically significant difference between the number and percentage of EQ(H)–SQ(L) participants in either group who selected the tournament (Fisher’s exact test with *p*-values adjusted according to the Benjamini and Hochberg method, *p* = 0.72, Odd’s ratio = 0.52; *t*-test with *p*-values adjusted according to Tukey’s method, *p* = 0.39, 95% CI = − 0.37 to 0.15, Cohen’s *d* = 0.31). Fourteen of the 46 EQ(L)–SQ(H) participants (30.4%) in the oxytocin group and 12 of the 47 EQ(L)–SQ(H) participants (25.5%) in the placebo group entered the tournament. However, there was no statistically significant difference between the two groups in the proportion of participants entering the tournament (Fisher’s exact test with *p*-values adjusted according to the Benjamini and Hochberg method, *p* = 1.00, Odd’s ratio = 1.28; *t*-test with *p*-values adjusted according to Tukey’s method, *p* = 0.96, 95% CI = − 0.06 to 0.42, Cohen’s *d* = − 0.11).

## Discussion

The current double-blinded investigation reveals that oxytocin does not affect competitiveness in men. When we divided our participants according to the presence or absence of autistic traits, we observed no relationship between oxytocin and competitiveness among participants with or without autistic traits, although our results show a suggestive pattern, in that oxytocin moderated competitiveness among participants without autistic traits.

Although we hypothesized that oxytocin moderates competitiveness, we found this not to be the case when we aggregated all participants. Cooperativity has been shown to be positively correlated with prosociality^30,31^ and negatively with competitiveness^28^. Based on these past studies, we thus hypothesized that oxytocin enhances prosociality and moderates competitiveness; however, our current findings indicate that this simple implication does not hold. On the other hand, our results also imply that oxytocin may affect competitiveness via different mechanisms in individuals with and without autistic traits. In fact, previous studies reported that competitiveness is a composite of several personality inventories^35^ or economically-relevant preferences^18^. One important factor is feedback aversion^18^. Competitions require participants to overcome tension or anxiety to accept their relative position. Kirsch^36^ reports, on the basis of functional magnetic resonance imaging (fMRI), that an intake of oxytocin reduced the activation of the amygdala and modulated the relevant network processing of fear, implying a role of oxytocin in the alleviation of fear. Such a mechanism may be predominant in those individuals with higher autistic-like traits, while oxytocin may promote prosociality in those with less autistic-like traits.

Our results are inconsistent with those of a prior study reporting that oxytocin enhances prosociality among individuals with less autistic-like traits^5^, but agree with the observation of another study that oxytocin does not improve the interpersonal communication skills of individuals with ASD^37^. As shown in Parker et al.^6^, the effect of oxytocin on prosociality depends on baseline oxytocin levels; this dependency may be more decisive than autistic-like traits.

One limitation of our study is that we did not measure oxytocin levels. Future investigations of whether baseline and subsequent changes in oxytocin levels affect competitiveness are therefore necessary. The present study was further limited by our restriction of the analysis to men. Considering that Yao et al.^38^ found oxytocin to induce prosocial behaviours in women but not men, future research should explore whether oxytocin has a negative effect on competitiveness in women. The need for such research is underscored by the specific role of oxytocin in female reproductive functions, such as in facilitating parturition and milk ejection during lactation^39,40^. If oxytocin does moderate competitiveness in women, an aversion to working in competitive environments may result from temporary incremental increases in oxytocin that could further affect long-term career selection and duration. Furthermore, relative to men, women reportedly shy away from competition: a tendency that may—at least in part—account for the gender gap in wage and career prospects^41^. In addition to oxytocin, sex hormones such as progesterone and oestrogen are female-specific hormones. One study^27^ showed that women with low progesterone and oestrogen levels avoid competition; however, subsequent observations demonstrated the opposite to be the case^20^. Finally, as the relationship between individual traits and competitiveness remains unclear, a better understanding of internal factors that influence competitiveness, such as psychological characteristics, is required.

In summary, we found no effect of oxytocin on competitiveness in men. Although we obtained null results, we identified the following trends: oxytocin may moderate competitiveness among individuals without autistic-like traits, and considering competitiveness the opposite of prosociality may be an inaccurate representation. These suggestive patterns will help improve our understanding of competitiveness. In addition, we believe that null results such as the current findings are meaningful in that they help avoid publication biases.

## Methods

### Participants

We conducted our experiment at Osaka University on October 4, 11, 13, and 25, and on November 1, 2017. All experimental protocols were approved by the ethics committee of the Institute of Social and Economic Research, Osaka University, where the experiment was conducted, and by the ethics committee of Kanazawa University Hospital, where oxytocin and placebo were prepared, dispensed, and measured. All experiments were performed in accordance with relevant guidelines and regulations. We ran nine sessions with 192 healthy, non-smoking, male students from Osaka University. Twenty participants were included in the first six sessions and 24 in the last three sessions. Participants were instructed not to eat or drink (anything other than water) for 2 h before the experiment and to abstain from alcohol and caffeine on the evening before the day of the experiment. Participants were also informed that oxytocin would be administered in this experiment. Informed consent for the experiment was obtained from all participants before the experiment started.

### Oxytocin administration

We used a double-blinded random control trial study design to compare competitiveness between participants who received oxytocin and those who were administered a placebo; participants were randomly assigned to either group. Subjects in the oxytocin group received a single intranasal dose of 24 IU oxytocin (Syntocinon-Spray, 3 puffs per nostril, each with 4 IU), and those in the placebo group received placebo (which contained chlorobutanol, methyl p-hydroxybenzoate, propyl p-hydroxybenzoate, and anhydrous citric acid). After administration of oxytocin or placebo, participants rested for approximately 10 min during which they were seated at a round table and could talk with other participants. The CE then began immediately after the instructions were presented to the participants. Overall, about 30 min passed until the beginning of the CE. When oxytocin diffuses into the brain and binds to oxytocin receptors, the neurons release oxytocin^42^. Recently, MRI blood flow monitoring of the human brain has shown that OT reaches various brain regions through unidentified nose-to-brain transport^43^. The same group also reported that nasal OT spreads via systemic circulation and is then taken from the blood to the amygdala^43^. In addition, recently, Yamamoto et al.^44^ identified a responsive molecule for OT brain transport: the receptor for advanced glycation end-products (RAGE) is an OT binding protein and plays a critical role in the transport of OT from the blood to the brain in mice^44,45^. These reports give us some validity for targeting central OT receptors after nasal OT administration.

### Competition experiment

The experimental design largely followed the framework depicted by Niederle and Vesterlund^18^. Each participant was assigned to a group of four; however, participants were blinded to the group assignment of the other participants. The experiment consisted of a practice session and four tasks. To measure the level of the participants’ effort, we used the slider task^46^, because it allows for the precise elicitation of the participant’s effort level without the influence of pre-existing knowledge^47^. The wide-spread adoption of the slider task in recent experimental studies in economics underscores its suitability for the present experiment^48–50^. Supplement Fig. 1 shows a screenshot of the participants’ display. There were 48 sliders, all of which were initially positioned at 0. The position of each slider was displayed to the right of the slider. Each slider could be adjusted to positions between the values of 0 and 100. Once the session started, participants were asked to move the slider to 50, which required delicate positioning with a mouse. The adjustment of one slider did not affect the position of the other sliders, and no two sliders were aligned so as to prevent the easy adjustment of the slider by referencing the adjacent sliders. Participants received 1 point if a slider was positioned exactly at 50. They could adjust the slider as many times as they wished within 90 s to achieve a position as close to 50 as possible. Participants successfully adjusted a mean of 19 sliders with a standard deviation of four sliders (Table 1). Prior to the actual experiment, participants were allowed to practice this slider task during two practice sessions, for which they received no compensation.

After completing the practice sessions, participants progressed on to the actual tasks. In Task 1, participants were paid on a piece-rate scheme: they received ¥50 for every point. For instance, if a participant earned 10 points, he received ¥500 (10 points × ¥50) regardless of other points earned by the group members. Task 2 employed a tournament payment scheme: the participant who earned the most points in each group received ¥200 for every point, while the others received ¥0. For instance, if the highest-scoring participant earned 10 points, he would receive ¥2000 (10 points × ¥200). However, if anyone had earned more points than him, his points would have not been compensated. Since the points earned under the tournament payment scheme depended on the performance of the opponents in the same group, participants were exposed to a competitive environment. In Task 3, the participants were asked to choose whether they wanted to be paid under a piece-rate or tournament scheme before beginning the slider task. The participants’ choice in Task 3 was then used as a measure of their competitiveness.

After completing Task 3, participants were asked to choose whether they wanted to be compensated under the piece-rate or the tournament scheme for their performance in Task 1 (Task 4). If participants chose the piece-rate scheme, they received ¥50 for every point in Task 1; if they chose the tournament scheme, they received ¥200 for every point if they were the best performer and ¥0 if they were not. The difference between Task 3 and 4 was that participants really performed the slider task in Task 3 and not in Task 4. The subjects’ choice in Task 4 was influenced by factors such as overconfidence, risk-aversion, feedback aversion, and performance level, but not by their preference for competition^18^. Conversely, the participant’s choice in Task 3 involved actual competition, as well as speculation on their relative performance. However, we did not report the results of Task 4, in order to focus on the participants’ choices in Task 3.

All participants received a ¥3000 show-up fee and were paid for one randomly selected task from the CE. Their reward was based on their choice to reveal their true preference. This is a standard incentivized method used in experimental economics. The experiment was programmed using z-Tree^51^.

### Statistical analysis

We set the significance level to 10% for all statistical tests used in this paper. We used two-sided t-tests to test our hypothesis that autistic-like traits (AQ, EQ, and SQ scores) and performance under the piece-rate and tournament schemes would not differ between the oxytocin and placebo groups. Further hypothesizing that oxytocin would reduce competitiveness—as measured by the participants’ selection in Task 3—we used one-sided Fisher’s exact tests and one-sided *t*-tests to compare competitiveness between the two groups. For comparisons of more than two groups, we used Fisher’s exact test for multiple-comparisons, with *p*-values adjusted according to the Benjamini and Hochberg method, and *t*-tests with *p*-values adjusted according to Tukey’s method.

With the exception of Fisher’s exact tests with *p*-values adjusted according to the Benjamini and Hochberg method that were conducted with R (R Foundation for Statistical Computing, Vienna, Austria), all statistical analyses were performed using Stata 13 (StataCorp, College Station, TX, USA). All Figures were created with R.

## Supplementary information

Supplementary Information 1.

## Data Availability

The computer code used in the experiment is available from the corresponding author on request.
