# Supplementary material for: Competitiveness and individual characteristics: a double-blind placebo-controlled study using oxytocin
Source: Sci Rep. 2020 Jul 13;10:11526. doi: 10.1038/s41598-020-68445-w (PMC7359354; doi:10.1038/s41598-020-68445-w)
Supplement: Supplementary file 1 — Supplementary Information 1. [file 41598_2020_68445_MOESM1_ESM.docx]

**Competitiveness and Individual Characteristics: A Double-Blind Placebo-Controlled Study Using Oxytocin**

Hirofumi Kurokawa^1^, Yusuke Kinari2, Hiroko Okudaira3, Kiyotaka Tsubouchi4, Yoshimichi Sai^4^, Mitsuru Kikuchi^4^, Haruhiro Higashida^4^, Fumio Ohtake5

^1^ School of Economics and Management, University of Hyogo, Kobe, Hyogo, Japan

^2^ Hirao School of Management, Konan University, Nishinomiya, Hyogo, Japan

^3^ Doshisha Business School, Doshisha University, Kyoto, Kyoto, Japan

^4^ Research Center for Child Mental Development, Department of Biophysical Genetics, Kanazawa University, Kanazawa, Kanazawa, Japan

^5^ Department of Economics, Osaka University, Toyonaka, Osaka, Japan

**Corresponding author**:

Hirofumi Kurokawa, School of Economics and Management, University of Hyogo

8-2-1 Gakuen-nishi-machi, Nishi-ku, Kobe, Hyogo 651-2197 Japan

+81-78-794-5412

kurokawa@em.u-hyogo.ac.jp

**Supplementary Figures**


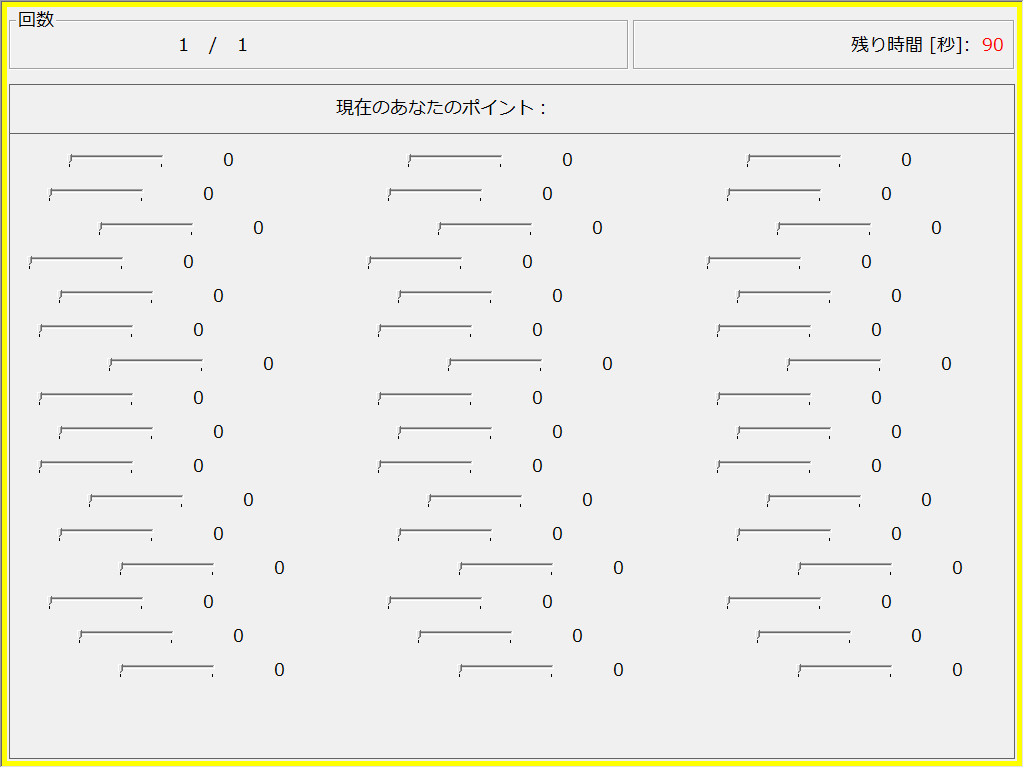


**Supplementary Figure 1. Screenshot of the slider task.** There were 48 sliders positioned at 0 for the first task. Each slider could be adjusted between values 0 and 100. Subjects received 1 point if a slider was positioned at 50. In this task, participants adjusted and readjusted sliders using a mouse for an unlimited number of times within 90 seconds.


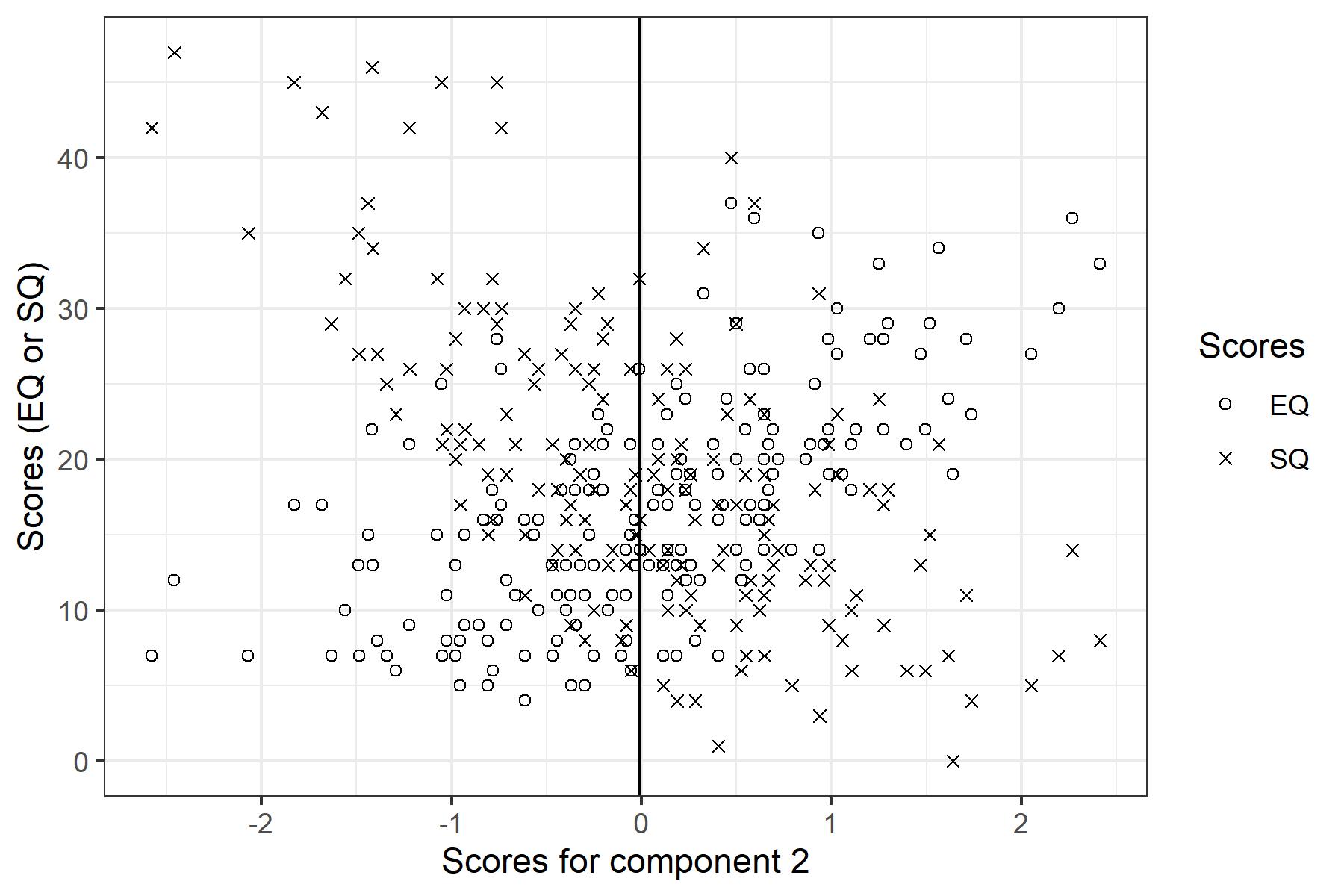


**Supplementary Figure 2. Scatterplot of scores for the second principal component of EQ-SQ and EQ or SQ.** The red vertical line indicates the median of scores for the second component (-0.01). The region on the left side of the median indicates EQ(L)-SQ(H) (i.e., Low-EQ and High-SQ). The region on the right side of the median indicates EQ(H)-SQ(L) (i.e., High-EQ and Low SQ). EQ, empathy quotient; SQ, systemizing quotient

**
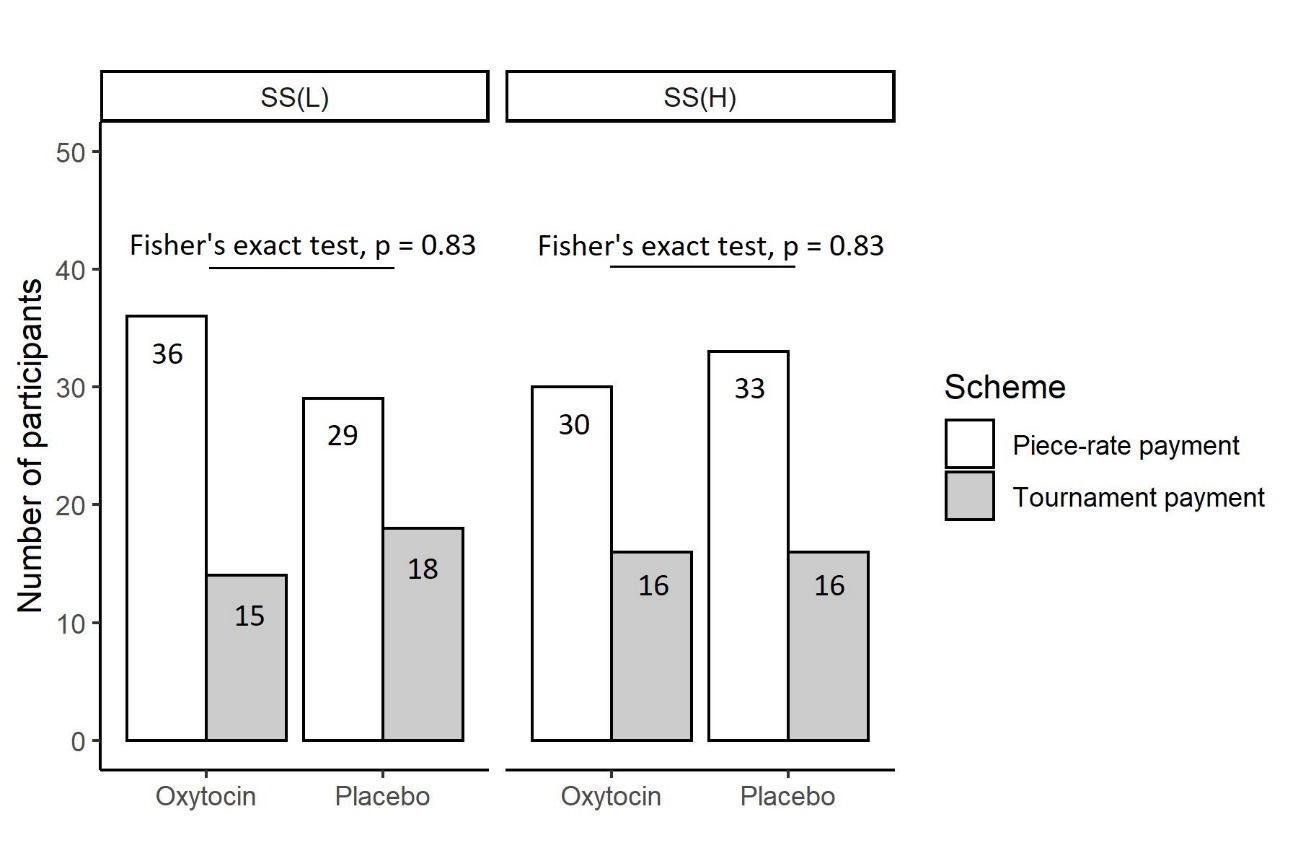
Supplement Figure 3. Number of participants choosing the piece-rate or tournament payment scheme in Task 3 according to treatment and social skill scores.** We assigned participants whose social skill scores were higher and lower than the median into the SS(H) and the SS(L) group, respectively. Fifteen of the 50 SS(L) participants in the oxytocin group and 18 of the 47 SS(L) participants in the placebo group chose the tournament payment scheme (Fisher’s exact test with p values adjusted according to the Benjamini and Hochberg method, p = 0.83). Sixteen of the 46 SS(H) participants in the oxytocin group and 16 of the 49 SS(H) participants in the placebo group chose the tournament payment scheme (Fisher’s exact test with p values adjusted according to the Benjamini and Hochberg method, p = 0.83).

**
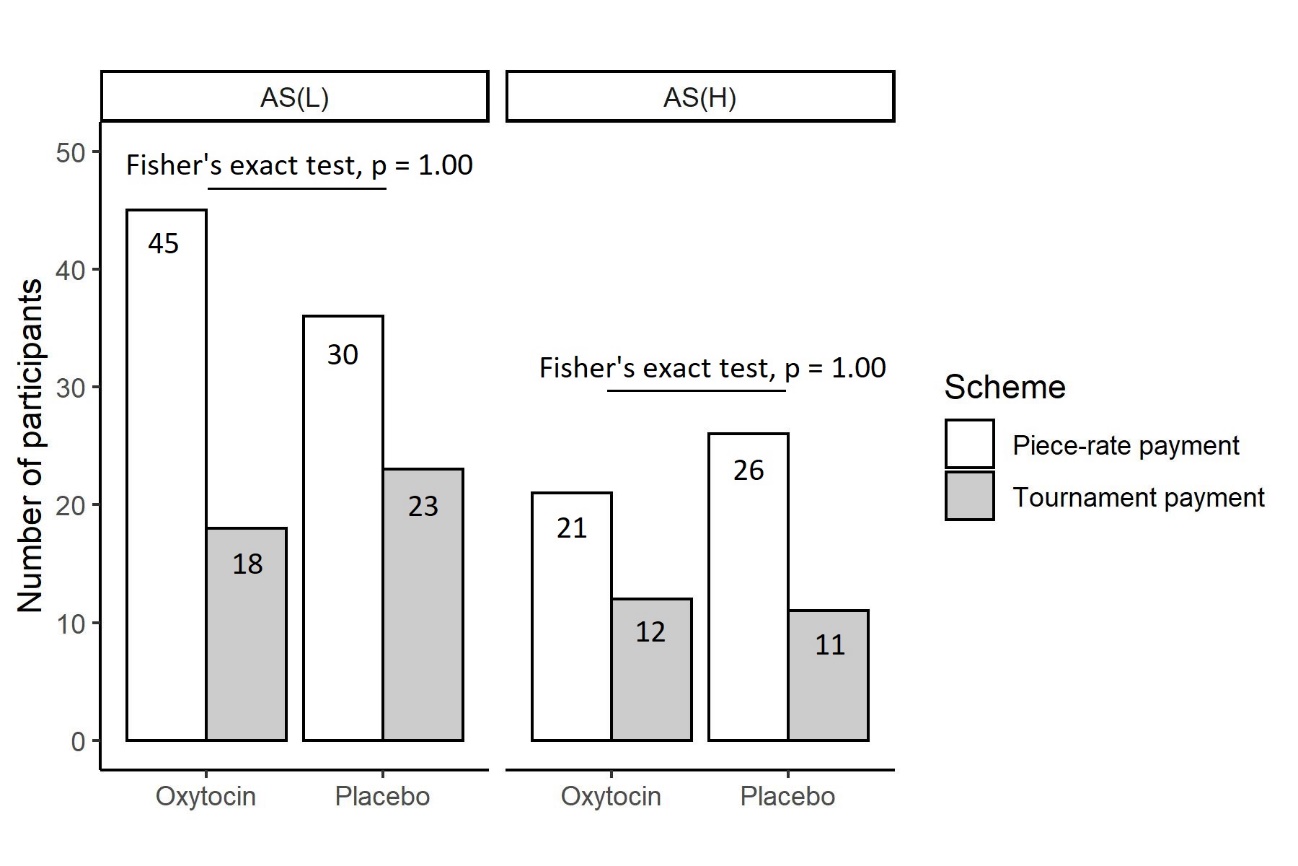
Supplement Figure 4. Number of participants choosing the piece-rate or tournament payment scheme in Task 3 according to treatment and attention switching scores.** We assigned participants whose attention switching scores were higher and lower than the median into the AS(H) and the AS(L) group, respectively. Eighteen of the 63 AS(L) participants in the oxytocin group and 23 of the 53 AS(L) participants in the placebo group chose the tournament payment scheme (Fisher’s exact test with p values adjusted according to the Benjamini and Hochberg method, p = 1.00). Twelve of the 33 AS(H) participants in the oxytocin group and 11 of the 37 AS(H) participants in the placebo group chose the tournament payment scheme (Fisher’s exact test with p values adjusted according to the Benjamini and Hochberg method, p = 1.00).


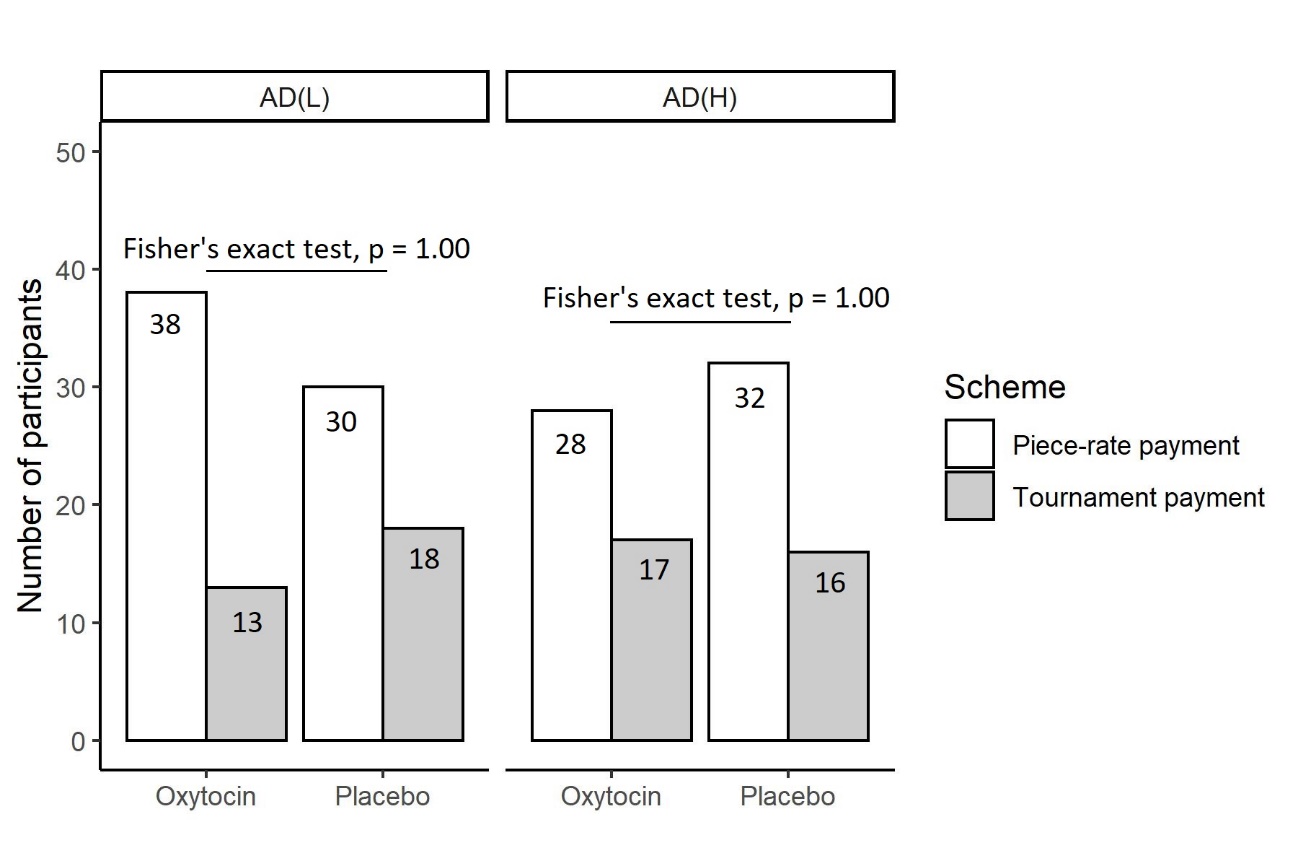


**Supplement Figure 5. Number of participants choosing the piece-rate or tournament payment scheme in Task 3 according to treatment and attention to detail scores.** We assigned participants whose attention to detail scores were higher and lower than the median into the AD(H) and the AD(L) group, respectively. Thirteen of the 51 AD(L) participants in the oxytocin group and 18 of the 48 AD(L) participants in the placebo group chose the tournament payment scheme (Fisher’s exact test with p values adjusted according to the Benjamini and Hochberg method, p = 1.00). Seventeen of the 45 AD(H) participants in the oxytocin group and 16 of the 48 AD(H) participants in the placebo group chose the tournament payment scheme (Fisher’s exact test with p values adjusted according to the Benjamini and Hochberg method, p = 1.00).


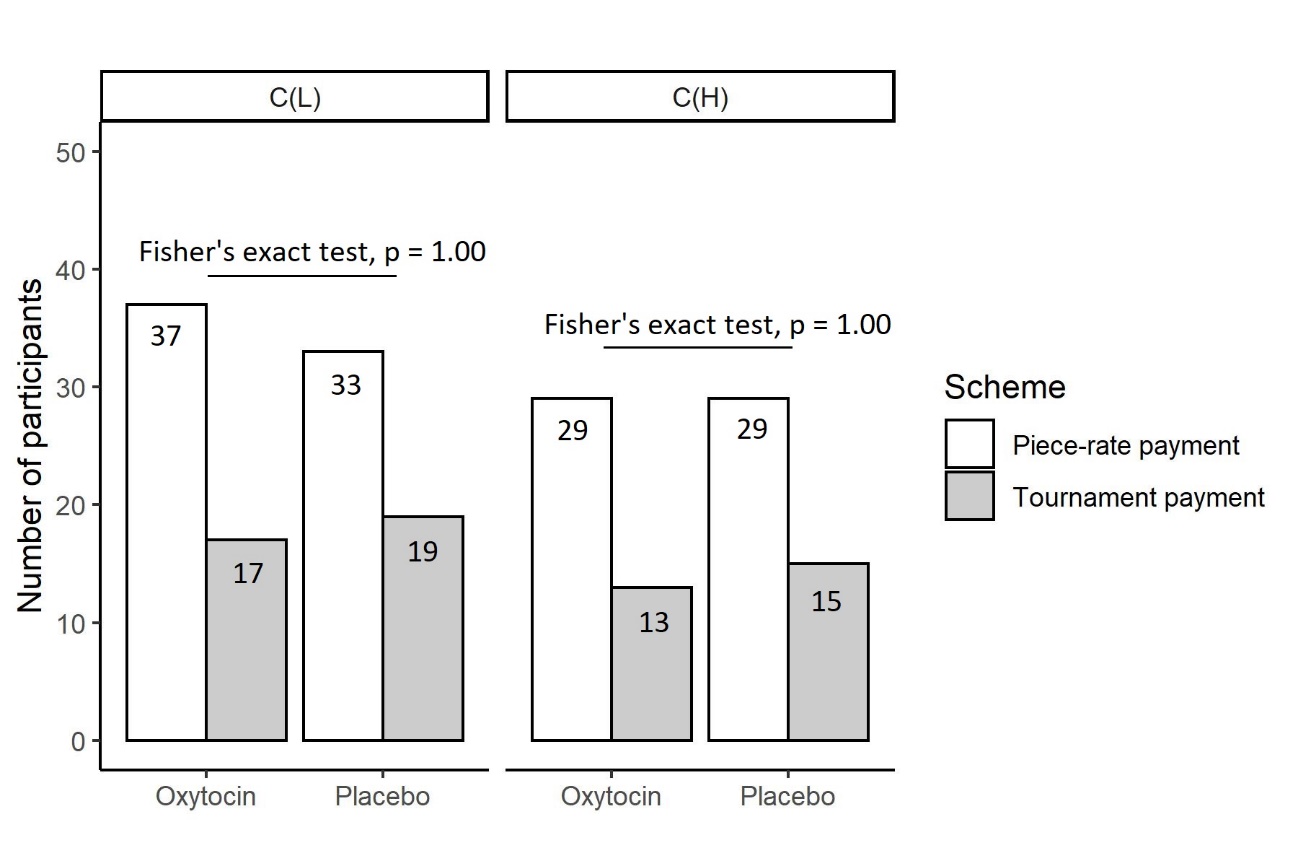


**Supplement Figure 6. Number of participants choosing the piece-rate or tournament payment scheme in Task 3 according to treatment and communication scores.** We assigned participants whose communication scores were higher and lower than the median into the C(H) and the C(L) group, respectively. Seventeen of the 54 C(L) participants in the oxytocin group and 19 of the 52 C(L) participants in the placebo group chose the tournament payment scheme (Fisher’s exact test with p values adjusted according to the Benjamini and Hochberg method, p = 1.00). Thirteen of the 42 C(H) participants in the oxytocin group and 15 of the 44 C(H) participants in the placebo group chose the tournament payment scheme (Fisher’s exact test with p values adjusted according to the Benjamini and Hochberg method, p = 1.00).


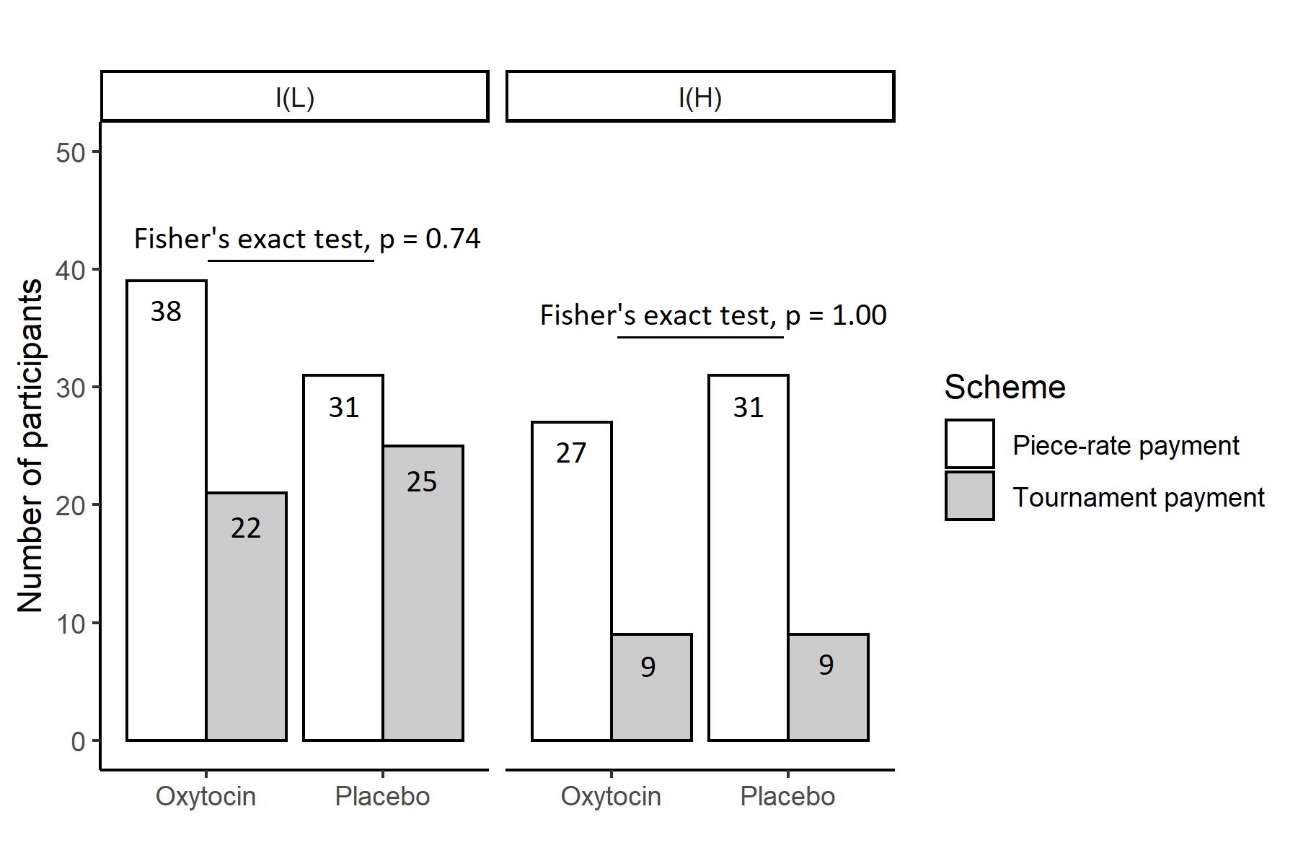


**Supplement Figure 7. Number of participants choosing the piece-rate or tournament payment scheme in Task 3 according to treatment and imagination scores.** We assigned participants whose imagination scores were higher and lower than the median into the I(H) and the I(L) group, respectively. Twenty-two of the 60 I(L) participants in the oxytocin group and 25 of the 56 I(L) participants in the placebo group chose the tournament payment scheme (Fisher’s exact test with p values adjusted according to the Benjamini and Hochberg method, p = 0.74). Nine of the 36 I(H) participants in the oxytocin group and nine of the 40 I(H) participants in the placebo group chose the tournament payment scheme (Fisher’s exact test with p values adjusted according to the Benjamini and Hochberg method, p = 1.00).

**Supplementary Tables**

**Supplement Table 1**. Summary statistics for the AQ subscale.

|  | All | | |  | Oxytocin | | |  | Placebo | | |  |
| --- | --- | --- | --- | --- | --- | --- | --- | --- | --- | --- | --- | --- |
|  | N | Mean | SD |  | N | Mean | SD |  | N | Mean | SD | p-values |
| Social skills | 192 | 2.59 | 1.97 |  | 96 | 2.61 | 1.98 |  | 96 | 2.57 | 1.98 | 0.88 |
| Attention switching | 192 | 3.01 | 1.34 |  | 96 | 3.03 | 1.28 |  | 96 | 2.99 | 1.40 | 0.83 |
| Attention detail | 192 | 1.44 | 0.95 |  | 96 | 1.47 | 0.98 |  | 96 | 1.41 | 0.92 | 0.65 |
| Communication | 192 | 3.22 | 2.17 |  | 96 | 3.23 | 2.06 |  | 96 | 3.22 | 2.29 | 0.97 |
| Imagination | 192 | 2.21 | 1.08 |  | 96 | 2.22 | 1.08 |  | 96 | 2.21 | 1.08 | 0.95 |
| Social skills (H) | 192 | 0.49 | 0.50 |  | 96 | 0.48 | 0.50 |  | 96 | 0.51 | 0.50 | 0.67 |
| Attention switching (H) | 192 | 0.36 | 0.48 |  | 96 | 0.34 | 0.48 |  | 96 | 0.39 | 0.49 | 0.55 |
| Attention detail (H) | 192 | 0.48 | 0.50 |  | 96 | 0.47 | 0.50 |  | 96 | 0.50 | 0.50 | 0.67 |
| Communication (H) | 192 | 0.45 | 0.50 |  | 96 | 0.44 | 0.50 |  | 96 | 0.46 | 0.50 | 0.77 |
| Imagination (H) | 192 | 0.40 | 0.49 |  | 96 | 0.38 | 0.49 |  | 96 | 0.42 | 0.50 | 0.56 |

Subscales of the AQ include social skills, attention switching, attention to detail, communication, and imagination. We generated dummy variables for each subscale, which equaled 1 if the subscale score was higher than the median. No AQ subscale differed significantly between the oxytocin and the placebo groups, indicating that the randomization assignment was successful.
